# A Swarming Approach to Optimize the One-Hop Delay in Smart Driving Inter-Platoon Communications

**DOI:** 10.3390/s18103307

**Published:** 2018-10-01

**Authors:** Qiong Wu, Shuzhen Nie, Pingyi Fan, Hanxu Liu, Fan Qiang, Zhengquan Li

**Affiliations:** 1 Jiangsu Provincial Engineering Laboratory of Pattern Recognition and Computational Intelligence, Jiangnan University, Wuxi 214122, China; qiongwu@jiangnan.edu.cn (Q.W.); shuzhennie@vip.jiangnan.edu.cn (S.N.); hanxuliu@stu.jiangnan.edu.cn (H.L.); 2 National Mobile Communications Research Laboratory, Southeast University, Nanjing 210096, China; 3 Department of Electronic Engineering, Tsinghua University, Beijing 100084, China; 4 Advanced Networking Lab., Department of Electrical and Computer Engineering, New Jersey Institute of Technology, Newark, NJ 07102, USA; qf4@njit.edu

**Keywords:** multi-platoon, inter-platoon communications, swarming, one-hop delay

## Abstract

Multi-platooning is an important management strategy for autonomous driving technology. The backbone vehicles in a multi-platoon adopt the IEEE 802.11 distributed coordination function (DCF) mechanism to transmit vehicles’ kinematics information through inter-platoon communications, and then forward the information to the member vehicles through intra-platoon communications. In this case, each vehicle in a multi-platoon can acquire the kinematics information of other vehicles. The parameters of DCF, the hidden terminal problem and the number of neighbors may incur a long and unbalanced one-hop delay of inter-platoon communications, which would further prolong end-to-end delay of inter-platoon communications. In this case, some vehicles within a multi-platoon cannot acquire the emergency changes of other vehicles’ kinematics within a limited time duration and take prompt action accordingly to keep a multi-platoon formation. Unlike other related works, this paper proposes a swarming approach to optimize the one-hop delay of inter-platoon communications in a multi-platoon scenario. Specifically, the minimum contention window size of each backbone vehicle is adjusted to enable the one-hop delay of each backbone vehicle to get close to the minimum average one-hop delay. The simulation results indicate that, the one-hop delay of the proposed approach is reduced by 12% as compared to the DCF mechanism with the IEEE standard contention window size. Moreover, the end-to-end delay, one-hop throughput, end-to-end throughput and transmission probability have been significantly improved.

## 1. Introduction

The study on autonomous vehicles has been one of the hottest topics in recent years. Many automobile manufacturers and academic institutions across the world have focused on developing autonomous vehicles. In 2010, Google X Lab developed the first driverless vehicle and tested it successfully in California [1]. In 2014, Baidu, teaming up with Bavarian Motor Work (BMW), started up an autonomous driving research project, and tested the developed autonomous vehicles on complicated roads in Beijing and Shanghai in 2015 [2]. As compared with traditional vehicles, autonomous vehicles have the following advantages: the road safety can be considerably improved, thus saving thousands of lives [3]; traffic jams can be mitigated, thus reducing the fuel consumption [3]; user experience can be enhanced by liberating people from a long time’s driving, enabling them to drink coffee, deal with their business and so on [4].

Platooning is an important management strategy for autonomous vehicles, which can reduce traffic congestion, conserve energy consumption, improve traffic safety and facilitate the management of autonomous vehicles [5]. With platooning strategy, autonomous vehicles can maintain a small constant distance between them on the common lane with a constant speed [6]. In this case, autonomous vehicles follow one after another in a queue and organize themselves as a set called a platoon. Each platoon consists of a leader vehicle, a tail vehicle, and some member vehicles. Specifically, the leader vehicle is the first vehicle, which controls the speed and driving direction of the platoon. The tail vehicle is the last vehicle within the platoon. The rest are the member vehicles. Note that both the leader vehicle and tail vehicle refer to backbone vehicles, while the member vehicles are called non-backbone vehicles.

**Figure 1:**
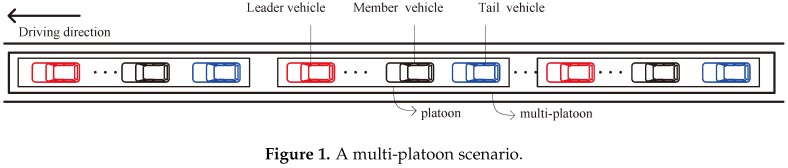
A multi-platoon scenario.

To keep a stable platoon formation, autonomous vehicles periodically transmit a packet with their kinematics information such as velocity and acceleration through vehicle-to-vehicle (V2V) communications [7]. Once the kinestate of a vehicle changes according to the surrounding environment, the vehicle, i.e., source vehicle, would transmit a packet with the updated kinematics information to other vehicles, i.e., destination vehicles. The destination vehicles can achieve the updated kinematics information in time and take prompt action to keep a stable platoon formation. When there are many vehicles in a platoon, the distance between the source vehicle and some destination vehicles may be long. The packet may be transmitted to some destination vehicles through multi-hop communications. The end-to-end delay would be long due to the plenty of collisions and hidden terminal problems caused by the simultaneous transmissions of many vehicles during the multi-hop communications. In this case, the packet delivery would suffer from the long end-to-end delay. Therefore, it is difficult to manage vehicles in a platoon especially when there are many vehicles [7]. To solve this problem, vehicles are organized into a multi-platoon instead of a single platoon [8]. A multi-platoon consists of a few platoons, as shown in Figure 1. In each platoon, the backbone vehicles are equipped with two transceivers [7]. One transceiver is used for inter-platoon communications, i.e., backbone vehicles communicate with other backbone vehicles. Another transceiver is used for intra-platoon communications, i.e., backbone vehicles communicate with the member vehicles in the same platoon. For a multi-platoon, the backbone vehicles in each platoon periodically transmit packets with their kinematics information to other backbone vehicles through inter-platoon communications, and then forward these packets to the member vehicles of the platoon through intra-platoon communications to keep a platoon formation.

For inter-platoon communications, backbone vehicles adopt the IEEE 802.11 distributed coordination function (DCF) mechanism to access a control channel (CCH) and communicate with each other through multi-hop communications [7]. There are several major factors that may affect the one-hop delay of inter-platoon communications:

1. The minimum contention window size defined in the IEEE 802.11 DCF mechanism. In fact, the IEEE 802.11 DCF mechanism uses the carrier sense multiple access with collision avoidance (CSMA/CA) mechanism to access a channel. It adopts a binary exponential back-off rule to reduce collisions. According to the IEEE 802.11 standard [9], the minimum contention window size is 64, which is too large. In this case, a vehicle would wait a lot of time during a back-off procedure for transmitting a packet. Owing to the long one-hop delay, the standard IEEE 802.11 DCF mechanism may not be suitable for inter-platoon communications.
2. The hidden terminal problem. For inter-platoon communications, if two backbone vehicles are not in the communication range of each other, packets transferred between them may arrive at a third backbone vehicle simultaneously, thus incurring a collision at the third backbone vehicle. This is the hidden terminal problem. One of the two backbone vehicles is the hidden terminal of another backbone vehicle. The hidden terminal problem would cause the two backbone vehicles retransmit the packet, resulting in the increased one-hop delay. In a multi-platoon, as the number of hidden terminals of each backbone vehicle is different, the one-hop delay of these backbone vehicles would be unbalanced.
3. The number of neighbors. The number of neighbors may be different for different backbone vehicles. The number of neighbors is 1 for the first and last backbone vehicle and 2 for the remaining backbone vehicles. When a backbone vehicle is transmitting a packet, if more than one neighbor is transmitting other packets at the same time, there would be a collision. In this case, the backbone vehicle has to retransmit the packet, and thus prolong the one-hop delay. For different backbone vehicles, if the number of neighbors is different, the collision probability would also be different and thus incurs an unbalanced one-hop delay.

As mentioned above, the parameters of DCF, the hidden terminal problem and the number of neighbors may affect the one-hop delay of inter-platoon communications and incur a long and unbalanced one-hop delay. It would affect the end-to-end delay from each source backbone vehicle to its destination backbone vehicles and make the destination backbone vehicles fail to receive vehicles’ kinematics information timely through inter-platoon communications. After a destination backbone vehicle receives vehicles’ kinematics information, it would forward the kinematics information to the member vehicles in the platoon through intra-platoon communications. Hence, a long and unbalanced one-hop delay of the inter-platoon communications cannot enable the member vehicles to obtain some emergency changes of vehicles’ kinestate within a limited time and take prompt action accordingly to keep a platoon formation. Therefore, the one-hop delay of inter-platoon communications is an important performance metric. To the best of our knowledge, there are no research works on optimizing the one-hop delay for inter-platoon communications. That motivates us to conduct this work.

In this paper, we propose a swarming approach to optimize the one-hop delay of inter-platoon communications by adjusting the minimum contention window size of each backbone vehicle in two steps. Then, we can get the adjusted minimum contention window sizes under different numbers of backbone vehicles in a multi-platoon. The performances such as one-hop delay, end-to-end delay, one-hop throughput, end-to-end throughput and transmission probability have been discussed by using the adjusted minimum contention window sizes.

The rest of this paper is organized as follows. Section 2 reviews the relevant recent works. The system model is introduced in Section 3. The swarming approach that optimizes the one-hop delay is described in Section 4. In Section 5, we discuss the network performance, and, finally, we conclude this paper in Section 6.

## 2. Related Work

This section introduces the relevant works. At first, the related works about the multi-platoon are surveyed; then, some related works that focus on the platoon without considering the impact of the multi-platoon are reviewed.

Only several works mainly focused on the performance of the multi-platoon. In [7], Peng et al. proposed a performance evaluation model considering the hidden terminal problem in a multi-platoon. They evaluated the performance in terms of throughput and packet delay from two aspects, i.e., the intra-platoon communications and inter-platoon communications. It has shown that the one-hop delay of the inter-platoon communications are unbalanced and relatively long. This inspired us to optimize the one-hop delay of inter-platoon communications. In [8], Fernandes and Nunes proposed a multi-platooning leaders positioning strategy and an algorithm to maintain a constant inter-platoon spacing and attain high traffic capacity. In [10], Peng et al. presented a sub-channel allocation scheme by jointly considering the broadcast services and device-to-device (D2D) communications. This scheme reduced the end-to-end delay and increased the successful delivery ratio in a multi-platoon. In [11], Harfouch et al. proposed a cooperative adaptive cruise control (CACC) strategy to achieve string stability in heterogeneous platoons and formulated a framework to evaluate the stability of the platoons. In [12], Ucar et al. proposed a hybrid safety message transmission protocol for multi-platoon which can overcome the low delivery ratio of safety application by adopting visible light communications (VLC). This protocol kept the advantage of 802.11p while guaranteeing the stability of a platoon and the security of inter-vehicle communications. In [13], Nardini et al. considered the sequential mode and simultaneous mode of dynamic scheduling when using the cellular vehicle-to-everything (C-V2X) communication technology, and proposed a resource allocation algorithm. The results have demonstrated that the simultaneous mode can reduce the data latency in high-density platoon scenarios.

Many works designed approaches to enhance the performance of the platoon without considering the impact of the multi-platoon. In [14], Guo and Li proposed a two-layer control system including a set-point optimization layer and a vehicle tracking control layer for platooning application. In the set-point optimization layer, Pontryagin’s minimum principle (PMP) was used to calculate the optimal speed set-point. In the vehicle tracking control layer, the string stability of vehicles for the platoons was guaranteed through a set of distributed sliding-mode controllers. In [15], Shen et al. proposed a dynamic platoon dispersion model which can be used to predict the evolution of traffic signal flows. The dynamic model considered the time-zone length, the percentages of turning, traffic flows and so on. In [16], Junior et al. proposed a platoon-based driving protocol which is called platoon-to-vehicle (P2V) based on game theory for video transmission with high quality of experience (QoE) support. The protocol combined some vehicles based on direction, speed and distance into a platoon. P2V improved the connectivity between these vehicles in a certain platoon and decreased the ratio of packet loss. In [17], Campolo et al. proposed a long-term evolution (LTE) system to support the additional V2V communication for data transmission in a platoon scenario. In [18], Besselink et al. proposed a delay-based and string stability spacing policy to determine the desired inter-vehicle spacing and enable all vehicles track the same path and velocity. In [19], Hoef et al. proposed an approach which formulated an optimal combination problem to solve the coordination of a large platoon and ensure low consumption of fuel. In [20], Wang et al. established a two-level system to detect the fault of the vehicle platoon. The system detected both system failure and component element failure. In [21], Wu et al. proposed a model to describe the behavior of the platoon. Based on the analysis results, the authors proposed an efficient control algorithm for the platoon based vehicular cyber-physical systems. In [22], Hu et al. proposed a TripSense scheme in a platoon trust-based system to perceive and process vehicles’ data. The data are collected by the member vehicles and aggregated by the head vehicle before they are transmitted to a server. In [23], Balador et al. proposed a beacon-based medium access control (MAC) protocol in a platoon. The simulations have demonstrated that the proposed protocol can enhance reliability and ensure the low-delay transmission for safety applications in intelligent traffic system (ITS).

From the related works mentioned above, we can see few works have considered the multi-platoon scenario while no studies have been conducted to optimize the one-hop delay of inter-platoon communications. A long and unbalanced one-hop delay of inter-platoon communication may affect the end-to-end delay and thus may pose a potential threat to safe driving, especially for a highway scenario. To solve this problem, we propose a swarming approach to reduce and balance the one-hop delay in inter-platoon communications.

## 3. System Model

For a multi-platoon, the backbone vehicles in platoons periodically transmit packets with vehicles’ kinematics information between them through inter-platoon communications, and then a backbone vehicle in a platoon forwards the packets to its platoon members through intra-platoon communications to keep a platoon formation. In this paper, we focus on the inter-platoon communications. In this section, we introduce the inter-platoon communications model.

**Figure 2:**
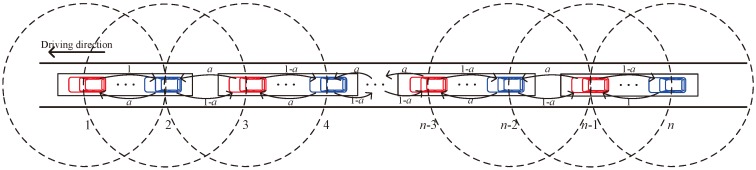
Inter-platoon communications model.

Considering a multi-platoon which is moving in a highway. Similar with [7], we assume that the inter-platoon spacing is not larger than the communication range of a backbone vehicle to guarantee the communication between the leader vehicle of a platoon and the tail vehicle of the preceding platoon. Moreover, the inter-platoon spacing is not smaller than the difference between the communication range of a backbone vehicle and the platoon length to avoid the collision between the leader (tail) vehicle in a platoon and the leader (tail) vehicle in the preceding platoon. In this case, there are one or two neighboring backbone vehicles within the communication range of a backbone vehicle. Specifically, the number of neighbor backbone vehicles is 1 for the first or last backbone vehicle of the multi-platoon and 2 for other backbone vehicles. Let *n* be the number of backbone vehicles in the multi-platoon. We label these backbone vehicles by 1, 2, ..., *n* − 1, *n*. For the inter-platoon communications, the backbone vehicles use the same transceiver to communicate with each other through multi-hop communications. The data traffic is saturated, i.e., a backbone vehicle always has packets to transmit. Let *a* be the probability that backbone vehicle *i* is the destination of backbone vehicle *i* + 1’s packets, where  1≤i<n−1. Hence, the probability that backbone vehicle *i* + 2 is the destination of backbone vehicle *i* + 1’s packet is 1 − *a*. The inter-platoon communications model is illustrated in Figure 2.

For the inter-platoon communications, backbone vehicles adopt the IEEE 802.11 DCF mechanism to access a CCH. When a backbone vehicle has a packet to transmit, it randomly selects an integer from [0, *W_k_* − 1] as the value of the back-off counter, here *k* denotes the number of the packet retransmission and *W_k_* is the minimum contention window size when the packet is retransmitted for *k* times. If the channel is detected to be idle, the value of the back-off counter would be decremented by one. Otherwise, the value of the back-off counter would be frozen until the channel keeps idle during a distributed inter-frame spacing (DIFS) period. Once the value of the back-off counter is decremented to 0, the backbone vehicle would transmit the packet. Afterwards, if the backbone vehicle does not receive an acknowledge (ACK) message after a short inter-frame space (SIFS) period, it would select an integer from [0, *W*_*k*+1_ − 1] randomly as the value of the back-off counter and initiate a new back-off procedure, in which *W*_*k*+1_ + 1 = 2*W_k_*. If the number of retransmissions reaches a retransmission limit, the backbone vehicles would drop this packet. In addition, to avoid the capture effect, a backbone vehicle should wait a random back-off value between two consecutive packet transmissions according to the back-off counter.

## 4. Swarming Approach Description

In this section, we propose a swarming approach to optimize the one-hop delay for inter-platoon communications in two steps, i.e., Section 4.1 and Section 4.2. In Section 4.1, a swarming approach is proposed to find a minimum average one-hop delay of inter-platoon communications by adjusting the minimum contention window of each backbone vehicle iteratively. In Section 4.2, the proposed swarming approach is used again to optimize the one-hop delay of each backbone vehicle to get close to the minimum average one-hop delay found in Section 4.1. After the second step, we can obtain the optimal minimum contention window sizes, i.e., the adjusted minimum contention window sizes that enable the one-hop delay of each backbone vehicle to get close to the minimum average one-hop delay.

### 4.1. Minimum Average One-Hop Delay

In Section 4.1, a swarming approach is proposed to find the minimum average one-hop delay of inter-platoon communications iteratively. In each iteration, the minimum contention window sizes of backbone vehicles are adjusted to enable the one-hop delays of backbone vehicles to get close to an optimization objective. The optimization objective is denoted as *D_avg_* that is usually set as a positive small value. In this paper, in order to obtain the minimum average one-hop delay, the value of *D_avg_* should be set as a value that the one-hop delay of each backbone vehicle cannot reach by adjusting the minimum contention window sizes. For simplicity, the value of *D_avg_* is set to be 0. After the optimization objective is determined, the minimum contention window sizes of backbone vehicles are adjusted to enable the one-hop delays of backbone vehicles to approach the optimal objective. The closeness between the one-hop delays of backbone vehicles and the optimization objective is evaluated by an objective function. The variance between the one-hop delays of backbone vehicles and *D_avg_* can be used to evaluate the closeness between them. Hence, the objective function is defined as the variance between the one-hop delays of backbone vehicles and *D_avg_*.

Next, we will introduce the swarming approach in detail. the swarming approach is divided into three sub-procedures, i.e., the initialization procedure, iteration procedure and output procedure. The pseudocode of the swarming approach is described as Algorithm 1.

#### 4.1.1. Initialization Procedure

As described at the beginning of Section 4.1, the optimization objective *D_avg_* is set to be 0. In the initialization procedure, *m* combinations of minimum contention window sizes are initialized. Each combination includes the minimum contention window sizes of *n* backbone vehicles. Each minimum contention window size is an integer randomly selected from [1, 64]. Here, *m* is an integer that cannot be too large or too small. If it is too large, the complexity of the swarming approach would be increased. If it is too small, the minimum average one-hop delay may not be found.

#### 4.1.2. Iteration Procedure

We first introduce the global optimal solution and the individual optimal solution of the swarming approach. In the iteration procedure, each combination including *n* minimum contention window sizes is updated iteratively to enable the one-hop delays of backbone vehicles to get close to *D_avg_*. Specifically, in each iteration, there is a global optimal solution for all combinations and an individual optimal solution for each combination. In the *t*th (*t* > 1) iteration, the global optimal solution *g*(*t*) is equal to the combination that minimizes the objective function value during the previous (*t* − 1)th iterations. The individual optimal solution of combination *j*, i.e., denoted as *p_j_*(*t*), is the combination that minimizes the objective function value of combination *j* during the previous (*t* − 1)th iterations. In the first iteration (*t* = 1), *g*(1) is the combination that minimizes the objective function value among all of the initial combinations and *p_j_*(1) is the initial value of combination *j*.

**Algorithm 1:** The Optimal Contention Window Sizes   1: *t* = 1;   2: Determine *D_avg_*;   3: **for**
j=1 to *m* (combinations) **do**   4:  **for**
i=1 to *n* (backbone vehicles) **do**   5:   initialize *cw_ij_*(*t*); // select randomly from [1, 64]   6:  **end for**   7: **end for**    //iteration procedure   8: **repeat**   9:  calculate *D_ij_*(*t*) through simulation;//Equation (1)  10:  **for**
j=1 to *m*
**do**  11:   *CW_j_*(*t*) = {*cw*_1*j*_(*t*), ..., *cw_ij_*(*t*), ..., *cw_nj_*(*t*)};  12:   calculate *f*(*CW_j_*(*t*)) under *D_avg_*;//Equation (2)  13:  **end for**     // update *gmin*(*t*) to the combination that     //minimizes the value of the objective function  14:  *gmin*(*t*) = *f*^−1^(min(*f*(*CW_j_*(*t*))));  15:  **if**
*t* == 1 **then**  16:   *g*(*t*) = *gmin*(*t*);  17:   *p_j_*(*t*) = *CW_j_*(*t*);  18:  **else**  19:   **if**
*f*(*gmin*(*t*)) < *f*(*g*(*t* − 1)) **then**  20:    *g*(*t*) = *gmin*(*t*);//Equation (3)  21:   **else**  22:    *g*(*t*) = *g*(*t* − 1);//Equation (3)  23:   **end if**  24:   **if**
*f*(*CW_j_*(*t*)) < *f*(*p_j_*(*t*-1)) **then**  25:    *p_j_*(*t*) = *CW_j_*(*t*);//Equation (4)  26:   **else**  27:    *p_j_*(*t*) = *p_j_*(*t*− 1);//Equation (4)  28:   **end if**  29:  **end if**  30:  **for**
j=1 to *m*
**do**  31:   **for**
i=1 to *n*
**do**  32:    compute Δ*cw_ij_*(*t*) ; //Equation (5)  33:    update *cw_ij_*(*t* + 1) ; //Equation (6), Equation (7)  34:   **end for**  35:  **end for**  36:  *t*++;  37: **until**
*f*(*g*(*t*)) < threshold or number of iterations == limit // output procedure  38: average *D_ij_*(*t*);  39: return the average result;

In addition, we briefly introduce the procedure of the swarming approach. For the first iteration, the inputs are the initial *m* combinations; the one-hop delays corresponding to each combination and the objective function value corresponding to each combination are calculated. Hence, the global optimal solution *g*(1) is determined by the combination corresponding to the minimum objective function value while the initial value of combination *j* is set as its the individual optimal solution *p*j**(1). If *g*(1) is smaller than a threshold, the iteration procedure would stop and the one-hop delays are the output. Otherwise, the related values of each combination is updated according to *g*(1), *p_j_*(1) and some parameters at the end of the first iteration. Similarly, for the *t*th (*t* > 1) iteration, the inputs are the *m* combinations updated at the end of the (*t* − 1)th iteration; the one-hop delays and the objective function value of each combination are calculated like the first iteration. Then, *g*(*t*) is determined by the combination with the minimum objective function value until the *t*th iteration, while *p_j_*(*t*) is set as the minimum objective function value of combination *j* until the *t*th iteration. In this case, if *g*(*t*) is smaller than a threshold or the number of iterations reaches the predefined maximum value, i.e., the iteration limit, the iteration would be stopped and the corresponding one-hop delays are the output. Otherwise, each combination is updated according to *g*(*t*), *p_j_*(*t*) and some parameters at the end of the *t*th iteration.

We will further analyze each iteration procedure in detail. For the *t*th iteration, the inputs are the *m* combinations updated at the end of the (*t* − 1)th iteration. Let *cw_ij_*(*t*) be the minimum contention window size of backbone vehicle *i* in combination *j*. In the system model, the backbone vehicle *i* in combination *j* transmits packets with the minimum contention window size *cw_ij_*(*t*). The one-hop delay of the backbone vehicle *i* in combination *j* is calculated by Equation (1),

(1) $$D_{ij}\left(t\right)=\frac{T_{ij}\left(t\right)}{x_{ij}\left(t\right)},$$

where *D_ij_*(*t*) is the one-hop delay of backbone vehicle *i* in combination *j*, *T_ij_*(*t*) is the time duration that backbone vehicle *i* in combination *j* takes to transmit packets, and *x_ij_*(*t*) is the number of the packets that are transmitted successfully by backbone vehicle *i* in combination *j* (Line 9).

Then, the closeness between the one-hop delays of the backbone vehicles with the minimum contention window sizes in combination *j* and *D_avg_* is measured through an objective function, i.e., the variance between them, which is given by (2),

(2) $$f\left(CW_{j}\left(t\right)\right)=\sum_{i=1}^{N}{\left[D_{ij}\left(t\right)-D_{avg}\right]}^{2},$$

where *CW_j_*(*t*) denotes the set of minimum contention window sizes of *n* backbone vehicles in combination *j*, i.e., *CW_j_*(*t*) = {*cw*_1*j*_(*t*), *cw*_2*j*_(*t*), ..., *cw_ij_*(*t*), ..., *cw_nj_*(*t*)}; *f*(*CW_j_*(*t*)) denotes the closeness between the one-hop delays of backbone vehicles with the minimum contention window sizes in combination *j* and *D_avg_* (Lines 10–13).

Given *m* combinations, the objective function values of them can be derived according to (2). The minimum objective function value among these combinations is *gmin*(*t*) (Line 14). Denote *gmin_i_*(*t*) as the minimum contention window size of backbone vehicle *i* in *gmin*(*t*), *g_i_*(*t*) as the minimum contention window size of backbone vehicle *i* in *g*(*t*), and *p_ij_*(*t*) as the minimum contention window size of backbone vehicle *i* in *p_j_*(*t*). When *t* > 1, *g_i_*(*t*) is selected through comparing *f*(*gmin*(*t*)) with *f*(*g*(*t* − 1)) and *p_ij_*(*t*) is selected through comparing *f*(*CW_j_*(*t*)) with *f*(*p_j_*(*t* − 1)) according to (3) and (4) respectively (Lines 19–28),

(3) $$g_{i}\left(t\right)=\left\{\begin{array}{cc} \;g_{i}\left(t-1\right), & f(gmin(t))\geq f(g(t-1)),t>1,\\ gmin_{i}\left(t\right), & f(gmin(t))<f(g(t-1)),t>1, \end{array}\right.$$

(4) $$p_{ij}\left(t\right)=\left\{\begin{array}{cc} \;p_{ij}\left(t-1\right), & f\left(CW_{j}\left(t\right)\right)\geq f\left(p_{j}\left(t-1\right)\right),t>1,\\ cw_{ij}\left(t\right), & f\left(CW_{j}\left(t\right)\right)<f\left(p_{j}\left(t-1\right)\right),t>1, \end{array}\right.$$

when *t* = 1, *g_i_*(*t*) is selected as *gmin_i_*(*t*) and *p_ij_*(*t*) is selected as *cw_ij_*(*t*)(Lines 15–17).

The basic idea of the above equations is to determine the one-hop delays by comparing how much the one hop delays of these vehicles are close to *D_avg_*. If *f*(*g*(*t*)) is smaller than a threshold value or the number of iterations reaches the iteration limit, the iteration procedure would be stopped and the one-hop delays of backbone vehicles that get close enough to the optimization objective *D_avg_* are obtained (Line 37).

If *f*(*g*(*t*)) is not smaller than a threshold value and the number of iterations does not reach the iteration limit, *cw_ij_*(*t*) would be updated according to *g*(*t*), *p_j_*(*t*) and some parameters at the end of the *t*th iteration. When *t* > 1, *cw_ij_*(*t*) is updated according to (5)–(7),

(5) $$\begin{array}{cc} \Delta cw_{ij}\left(t\right) & =w\cdot \Delta cw_{ij}\left(t-1\right)+c1\cdot r1\cdot \left(g_{i}\left(t\right)-cw_{ij}\left(t\right)\right)\\ & +c2\cdot r2\cdot (p_{ij}\left(t\right)-cw_{ij}\left(t\right)), \end{array}$$

(6) $$cw_{ij}^{\prime }\left(t+1\right)=cw_{ij}\left(t\right)+\Delta cw_{ij}\left(t\right),$$

(7) $$cw_{ij}\left(t+1\right)=\left[cw_{ij}^{\prime }\left(t+1\right)+0.5\right],$$

where *c*1 and *c*2 are the learning coefficients and are positive constants, *r*1 and *r*2 are the random parameters in [0, 1], *w* is the inertia coefficient, Δ*cw_ij_*(*t*) is the variation of *cw_ij_*(*t*), (7) is used to guarantee that the value of the updated *cw_ij_*(*t*) is the integer that equals the rounded-off value of $cw_{ij}^{\prime }\left(t+1\right)$. Here, Δ*cw_ij_*(*t*) cannot be larger than a constant Δ*cw_max_*. If Δ*cw_ij_*(*t*) is larger than Δ*cw_max_*, it would keep Δ*cw*_max_. When *t* = 1, Δ*cw*${}_{ij}$(*t*) is a random value ranging from 0 to 1, and *cw_ij_*(*t* + 1) is updated according to (6) and (7) (Lines 30–35).

#### 4.1.3. Output Procedure

In the output procedure, the inputs are the one-hop delays obtained from the proposed approach, i.e., the one-hop delays of backbone vehicles that get close enough to the optimization objective *D_avg_*. The minimum average one-hop delay is calculated by averaging the obtained one-hop delays. Finally, the minimum average one-hop delay are achieved.

**Figure 3:**
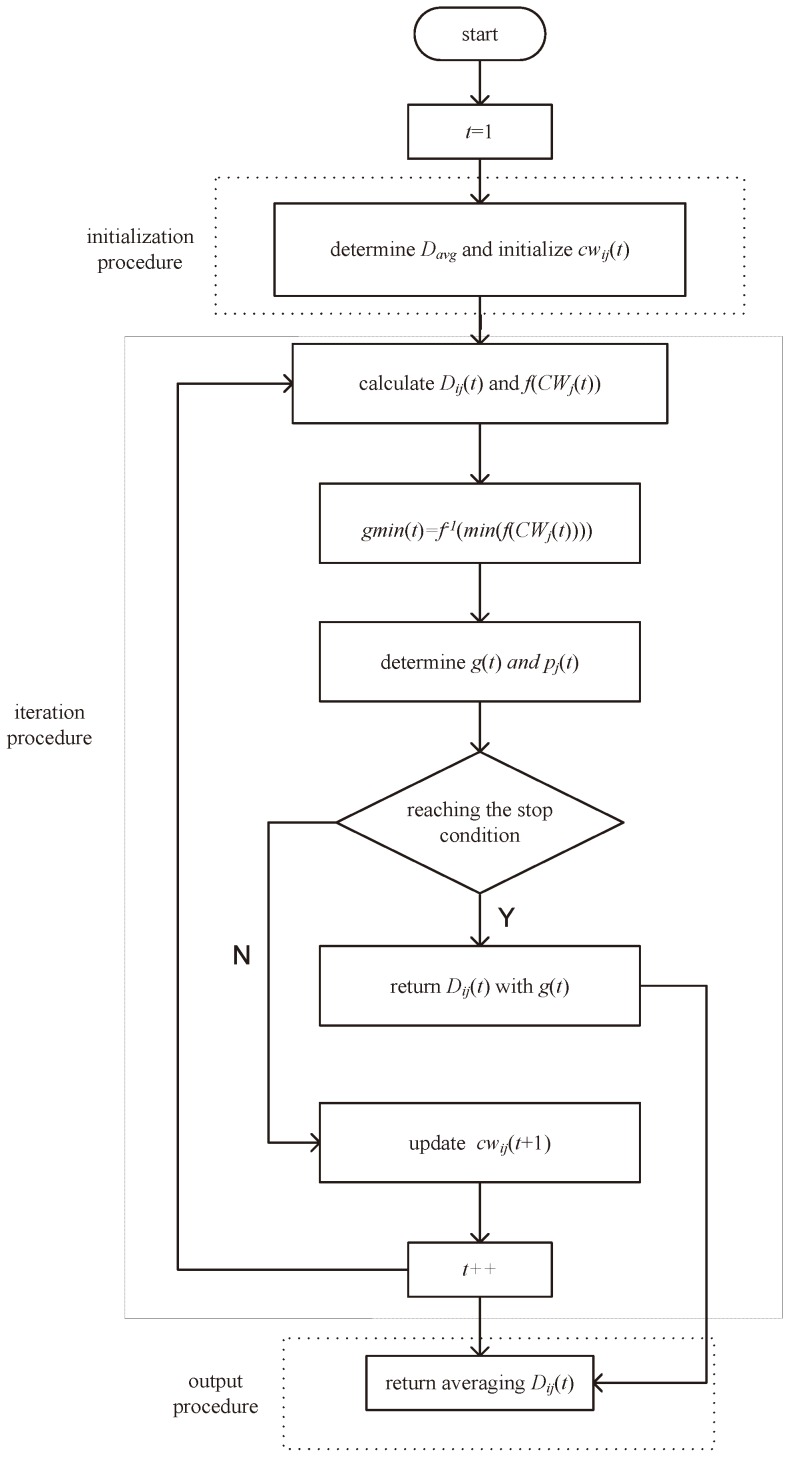
The flow chart of the swarming approach.

The flow chart of the swarming approach are described as Figure 3.

### 4.2. Optimal Minimum Contention Window Sizes

After Section 4.1, we have obtained the minimum average one-hop delay. Section 4.1 can guarantee that the one-hop delays of backbone vehicles get close to the optimization objective *D_avg_*, which is set to be 0 for simplicity. However, this does not guarantee that the one-hop delays get close to the minimum average one-hop delay. In Section 4.2, the optimal minimum contention window sizes of backbone vehicles are found, with which the one-hop delays of backbone vehicles could get close to the minimum average one-hop delay.

In Section 4.2, we aim to determine the optimum minimum contention window sizes of backbone vehicles by comparing how much the one-hop delays of these vehicles are close to the minimum average one-hop delay. As a result, the optimization objective is the minimum average one-hop delay and the objective function is to minimize the variance between the one-hop delays of backbone vehicles and the minimum average one-hop delay. The swarming approach proposed in Section 4.1 is used again to optimize the one-hop delay of each backbone vehicle to get close to the minimum average one-hop delay by adjusting the minimum contention window size. Finally, the outputs of Section 4.2 are the optimal minimum contention window sizes with which the one-hop delay of each backbone vehicle could get close to the minimum average one-hop delay.

## 5. Performance Results

In this section, we compare the performance in terms of the transmission probability, one-hop delay, end-to-end delay, one-hop throughput, end-to-end throughput and transmission probability of the inter-platoon communications under the optimal minimum contention window sizes and the standard minimum contention window size defined in the IEEE 802.11 DCF mechanism through simulation experiments. The simulation experiments were conducted using MATLAB (R2015a-academic use, 64 bit, MathWorks, Natick, MA, USA). The simulation codes are available in the supplementary material. The simulation scenario is described in Section 3. Let *CW_min_* be the standard minimum contention window size defined in the IEEE 802.11 DCF mechanism, *E*[*L*] be the size of each packet, *s* be the duration of a slot, *M* be the retransmission limit, *R* be the channel bit rate, *P_e_* be transmission error probability caused by channel, and *I_m_* be the iteration limit. Table 1 gives the parameters used in the simulation experiments.

**Figure 4:**
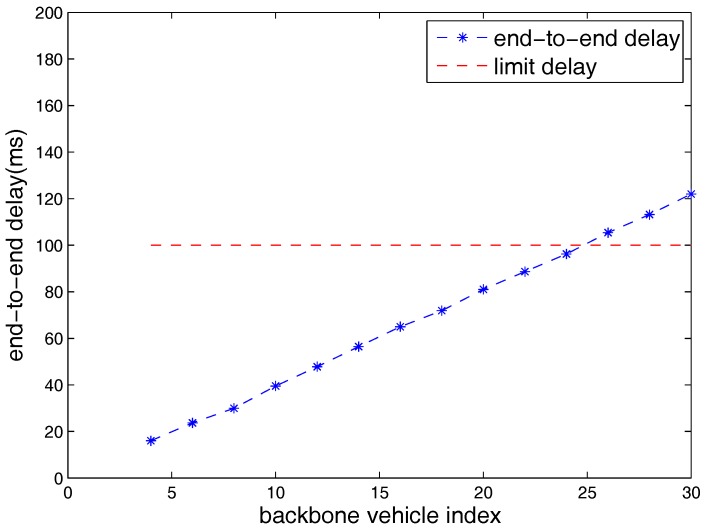
End-to-end delay vs. number of backbone vehicles.

Figure 4 shows the relationship between the end-to-end delay from the first backbone vehicle to the last backbone vehicle and the number of backbone vehicles in a multi-platoon when the standard minimum contention window size is used. In [24], European Telecommunications Standard Institute (ETSI) defined that a packet should be ensured to be accepted successfully within a maximum delay limit 100 ms. In [25], it also claimed that the maximum highway platooning end-to-end delay required for the ETSI and the 3rd Generation Partnership Project (3GPP) is 100 ms. In [26,27], they indicated that the most stringent required end-to-end latency is less than 100 ms and in the case of the small latency (less than 100 ms) the longitudinal and lateral error between the vehicles are low. In [28], the results show that most of the vehicle Central Address Memories (CAMs) have a delay of less than 2 ms when only inter-platoon communications exists. However, the corresponding experiments in [28] did not consider the backoff procedure of the IEEE 802.11 MAC protocol. As mentioned above, *in most related work*, the maximum end-to-end delay is 100 ms. Figure 4 illustrates that that the maximum number of backbone vehicles in a multi-platoon is 24 when the maximum delay is 100 ms.

The optimal minimum contention window sizes under the different number of backbone vehicles are given by Table 2. As there are two backbone vehicles in each platoon, the number of backbone vehicles varies from 4 to 24.

**Figure 5:**
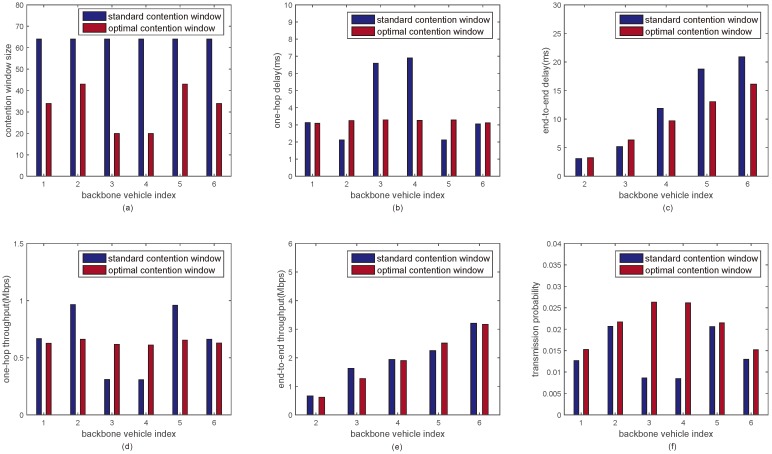
The performance of inter-platoon communications under six backbone vehicles. (**a**) Minimum contention window size vs backbone vehicle index; (**b**) One-hop delay vs backbone vehicle index; (**c**) End-to-end delay vs backbone vehicle index; (**d**) One-hop throughput vs backbone vehicle index; (**e**) End-to-end throughput vs backbone vehicle index; (**f**) Transmission probability vs backbone vehicle index.

In Figure 5a, the performance metrics such as the optimal minimum contention window size, the one-hop delay, end-to-end delay, one-hop throughput, end-to-end throughput and transmission probability with the optimal minimum contention window sizes are discussed respectively under six backbone vehicles. Figure 5a shows the comparison between the optimal minimum contention window size of each backbone vehicle and the standard minimum contention window size when the number of backbone vehicle is 6. It is seen that all optimal minimum contention window sizes are smaller than the standard minimum contention window size, thus it can guarantee a relatively low one-hop delay. Moreover, the minimum contention window size of each backbone vehicle is different to get the one-hop delay of each backbone vehicle balanced.

Figure 5b shows the comparison of the one-hop delay of each backbone vehicle between the proposed approach and the DCF mechanism with the standard minimum contention window size when the number of backbone vehicles is 6. It can be seen that the one-hop delays of backbone vehicles with the standard minimum contention window size are unbalanced. The one-hop delays of backbone vehicles of the proposed approach are balanced and kept at around a small value 3.2 ms.

Figure 5c shows the comparison of the end-to-end delay from backbone vehicle 1 to backbone vehicle *i* (*i* = 2, 3, ..., 6) between the proposed approach and the DCF mechanism with the standard minimum contention window size when the number of backbone vehicles is 6. It can be seen that the end-to-end delay from backbone vehicle 1 to backbone vehicle 6 with the optimal minimum contention window sizes is around 16 ms. This is around 5 ms lower than that with the standard minimum contention window size. Moreover, the end-to-end delays from backbone vehicle 1 to other backbone vehicles with the optimal minimum contention window sizes are increased slowly with the increasing of backbone vehicle index. This is because one-hop delay of each backbone vehicle with the optimal minimum contention window size is balanced.

Figure 5d shows the comparison of the one-hop throughput of each backbone vehicle between the proposed approach and the DCF mechanism with the standard minimum contention window size when the number of backbone vehicles is 6. It is seen that the one-hop throughput of each backbone vehicle with the optimal minimum contention window size is balanced and kept at around 0.6 Mbp/s.

Figure 5e shows the comparison of the end-to-end throughput from backbone vehicle 1 to backbone vehicle *i* (*i* = 2, 3, ⋯, 6) between the proposed approach and the DCF mechanism with the standard contention window size when the number of backbone vehicles is 6. It is seen that the end-to-end throughputs from backbone vehicle 1 to backbone vehicle 6 with the optimal minimum contention window sizes are around 3.2 Mbp/s. This is almost the same with the end-to-end throughputs by using the standard minimum contention window size. Moreover, the end-to-end throughputs from backbone vehicle 1 to other backbone vehicles with the optimal minimum contention window sizes are increased by a constant with the increasing of backbone vehicle index. This is because that the one-hop throughput of each backbone vehicle with the optimal minimum contention window size is balanced.

Figure 5f shows the comparison of the transmission probability of each backbone vehicle between the proposed approach and the DCF mechanism with the standard minimum contention window size when the number of backbone vehicles is 6. It is seen that the transmission probability of each backbone vehicle with the optimal minimum contention window size is larger than that with the standard minimum contention window size.

**Figure 6:**
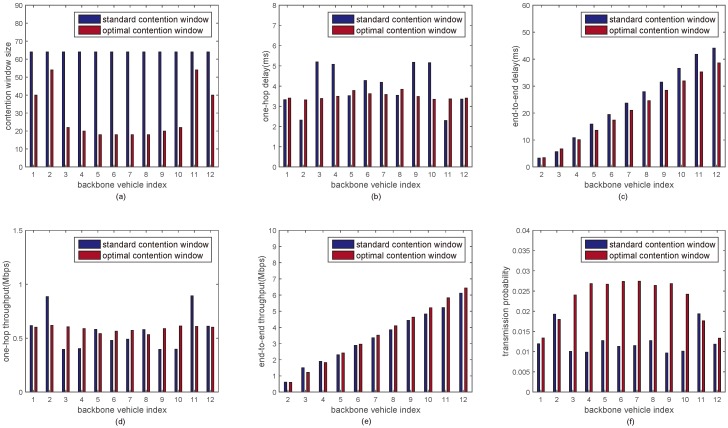
The performance of inter-platoon communications under 12 backbone vehicles. (**a**) Minimum contention window size vs backbone vehicle index; (**b**) One-hop delay vs backbone vehicle index; (**c**) End-to-end delay vs backbone vehicle index; (**d**) One-hop throughput vs backbone vehicle index; (**e**) End-to-end throughput vs backbone vehicle index; (**f**) Transmission probability vs backbone vehicle index.

**Figure 7:**
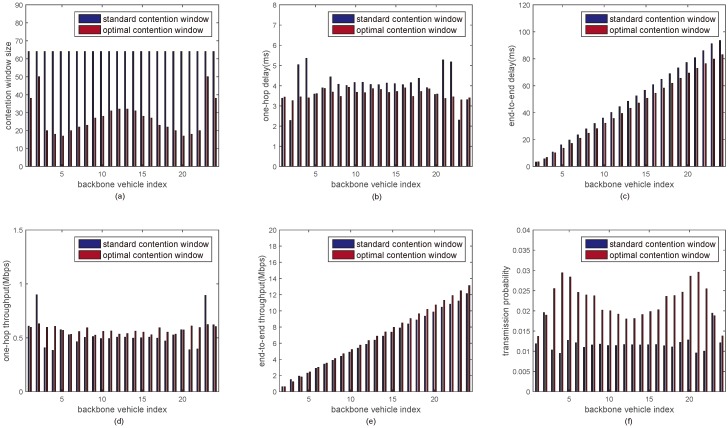
The performance of inter-platoon communications under 24 backbone vehicles. (**a**) Minimum contention window size vs backbone vehicle index; (**b**) One-hop delay vs backbone vehicle index; (**c**) End-to-end delay vs backbone vehicle index; (**d**) One-hop throughput vs backbone vehicle index; (**e**) End-to-end throughput vs backbone vehicle index; (**f**) Transmission probability vs backbone vehicle index.

In Figure 6 and Figure 7, the performance metrics with the optimal minimum contention window sizes are discussed under 12 and 24 backbone vehicles, respectively. Figure 6 and Figure 7 compare the performance of inter-platoon communications between the proposed approach and the DCF mechanism with standard minimum contention window size when the number of backbone vehicles in a multi-platoon is 12 and 24, respectively. It is seen that the one-hop delay and the one-hop throughput of each backbone vehicle with the optimal minimum contention window size is kept around a constant. The end-to-end delay from backbone vehicle 1 to backbone vehicle *i* (*i* = 2, 3, ⋯, 12/24) with the optimal minimum contention window sizes is reduced by 12%. The end-to-end throughput is increased by around 8% and the transmission probability is increased by about 40%.

**Figure 8:**
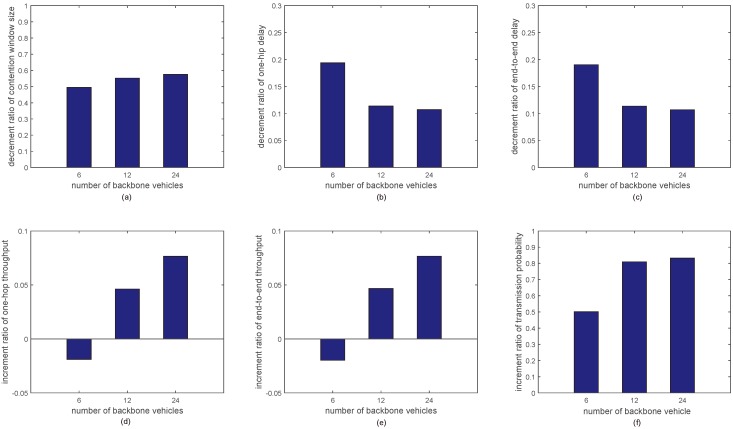
The average improvement ratio of the proposed approach’s performance under different numbers of backbone vehicles. (**a**) Decrement ratio of contention window size vs number of backbone vehicles; (**b**) Decrement ratio of one-hip delay vs number of backbone vehicles; (**c**) Decrement ratio of end-to-end delay vs number of backbone vehicles; (**d**) Increment ratio of one-hop throughput vs number of backbone vehicles; (**e**) Increment ratio of end-to-end throughput vs. number of backbone vehicles; (**f**) Increment ratio of transmission probability vs number of backbone vehicles.

Figure 8 shows the average improvement ratio of the proposed approach’s performance under different number of backbone vehicles. The average decrement ratio $\eta_{de}$ in Figure 8a–c is calculated by (8)

(8) $$\eta_{de}=\frac{M_{DCF}-M}{M_{DCF}},$$

and the average increment ratio $\eta_{in}$ in Figure 8d–f is calculated by (9)

(9) $$\eta_{in}=\frac{M-M_{DCF}}{M_{DCF}},$$

where *M* and $M_{DCF}$ are the summation of the metrics with the proposed approach and the DCF mechanism, respectively.

Figure 8a shows the average decrement ratio of the contention window size. It is seen that the optimal minimum contention window sizes are reduced by 49.4%, 55.2% and 57.6% as compared to the standard minimum contention window size when the number of backbone vehicles are 6, 12 and 24, respectively. The average decrement ratio of the contention window size increases with the increment of the number of backbone vehicles. Moreover, the decrement ratios of the contention window size under 12 and 24 backbone vehicles are almost the same. This means that the increment of the contention window size with the proposed approach is limited with the increment of the number of backbone vehicles.

Figure 8b,c shows the average decrement ratio of the one-hop delay and the end-to-end delay. It is seen that the one-hop delay and end-to-end delay are reduced by 19.4%, 11.4% and 10.7% as compared to the DCF mechanism when the number of backbone vehicles are 6, 12 and 24, respectively. The average decrement ratio of the one-hop delay and the end-to-end delay increase with the increment of the number of backbone vehicles.

Figure 8d,e shows the average increment ratio of the one-hop throughput and end-to-end throughput. It is seen that the one-hop throughput and end-to-end throughput are increased by −1.9%, 4.6% and 7.7% as compared to the DCF mechanism when the number of backbone of vehicles are 6, 12 and 24, respectively. Note that the average increment ratio of the one-hop delay and the end-to-end delay is a small negative value when the number of backbone vehicles is 6. However, as shown in Figure 5d,e, the one-hop throughput of each backbone vehicle with the optimal minimum contention window size is balanced under six backbone vehicles and the end-to-end throughputs from backbone vehicle 1 to other backbone vehicles with the optimal minimum contention window sizes are increased by a constant with the increasing of backbone vehicle index. Moreover, from Figure 8b,c, we can see that the one-hop delay and end-to-end delay of the proposed approach are decreased considerably under six backbone vehicles.

Figure 8f shows the average increment ratio of transmission probability. It is seen that the transmission probabilities are increased by 50.1%, 80.9% and 83.3% compared with the DCF mechanism when the number of backbone vehicles are 6, 12 and 24, respectively. The similar phenomenon appears as in Figure 8a. That is, the increment ratios of the transmission probability under 12 and 24 backbone vehicles are almost the same.

## 6. Conclusions

In this paper, we proposed a swarming approach to optimize the one-hop delay of inter-platoon communications through adjusting the minimum contention window size of each backbone vehicle in two steps. In the first step, we set a small average one-hop delay of backbone vehicles as the initial optimization objective and then proposed a swarming approach to find a minimum average one-hop delay of inter-platoon communications through adjusting the minimum contention window of each backbone vehicle iteratively. In the second step, we first set the minimum average one-hop delay found in the first step as the initial optimization objective and then adopt the swarming approach again to optimize the one-hop delay of each backbone vehicle to get close to the minimum average one-hop delay. After the second step, we can obtain the optimal minimum contention window sizes, i.e., the adjusted minimum contention window sizes with which the one-hop delay of each backbone vehicle could get close to the minimum average one-hop delay. The simulation results indicated that the one-hop delay is optimized and the other performance metrics including end-to-end delay, one-hop throughput, end-to-end throughput and transmission probability are considerably improved by using the optimal minimum contention window sizes, as compared with the IEEE standard contention window size.

## Figures and Tables

**Table 1:** Parameters used in the simulation experiments.

Parameter	Value	Parameter	Value
$CW_{min}$	64	*a*	0.15
E[L](bits)	2048	$P_{e}$	0.1
SIFS(us)	28	*m*	15
DIFS(us)	54	$c_{1}$	1.5
ACK(bits)	240	$c_{2}$	1.5
s(us)	13	*w*	0.8
*M*	5	$\Delta cw_{max}$	10
R(Mbps)	3	$I_{m}$	300

**Table 2:** The optimal contention window sizes.

*n*	The Optimal Minimum Contention Window Sizes
4	[38,49,49,38]
6	[34,43,20,20,43,34]
8	[38,55,24,22,22,24,55,38]
10	[36,51,22,20,18,18,20,22,51,36]
12	[40,54,22,20,18,18,18,18,20,22,54,40]
14	[32,45,18,18,17,20,20,20,20,17,18,18,45,32]
16	[44,56,23,18,16,17,20,21,21,20,17,16,18,23,56,44]
18	[34,45,17,15,14,15,16,17,18,18,17,16,15,14,15,17,45,34]
20	[34,50,21,24,23,30,29,30,27,26,26,27,30,29,30,23,24,21,50,34]
22	[42,55,19,15,13,14,17,19,22,21,21,21,21,22,19,17,14,13,15,19,55,42]
24	[38,50,20,18,17,20,22,23,27,28,31,32,32,31,28,27,23,22,20,17,18,20,50,38]

## Supplementary Materials

Simulation codes are available online at https://codeocean.com/2018/06/28/code-for-colon-a-swarming-approach-to.
